# The Controversy on Septicæmia

**Published:** 1873-09

**Authors:** 


### The Controversy on Septicæmia.

After a long interruption, the French Academy of Medicine has again had the subject of septicæmia brought under its notice by Professor VULPIAN, who has communicated the results of the numerous experiments he has undertaken in his labaratory, and which he thus summarises:—1. When rabbits or guinea-pigs are inoculated with the blood of a human being who has died of gangrene of the lung, and consequent septicæmia, they soon die, and their blood can prove, when injected into other animals, just as rapidly fatal. 2. To produce fatal results with blood which has been allowed to putrefy spontaneously and by exposure to the air, it is necessary to inject a considerable quantity, as much, in fact, as a cubic centimetre. The blood of the animal thus poisoned, however, acquires very virulent properties, and a much smaller quantity proves effective. 3. In certain cases the animals die at the expiration of several weeks, and the lesions consequent on purulent infection could then be demonstrated. 4. Blood taken from those affected with typhus fever never produced septicæmia in rabbits or guinea-pigs. 5. Well-marked differences in the characters of bacteria and vibrios are demonstrable in different instances, and VULPIAN thinks these differences may possess an important influence on the activity of infection. 6. Vulpian regards true pathological septicæmia as distinct from that produced by experiment, and suggests the name bacteræmia for the latter.—*Lancet.*

